# Outer Acoustic Streaming Flow Driven by Asymmetric Acoustic Resonances

**DOI:** 10.3390/mi13010065

**Published:** 2021-12-30

**Authors:** Junjun Lei, Gaokun Zheng, Zhen Yao, Zhigang Huang

**Affiliations:** 1 State Key Laboratory of Precision Electronic Manufacturing Technology and Equipment, Guangdong University of Technology, Guangzhou 510006, China; 2112101455@mail2.gdut.edu.cn (G.Z.); huangzg@gdut.edu.cn (Z.H.); 2 Guangzhou Key Laboratory of Non-traditional Manufacturing Technology and Equipment, Guangdong University of Technology, Guangzhou 510006, China; yaozhen@gdut.edu.cn

**Keywords:** acoustic streaming, boundary-driven streaming, asymmetric resonance, acoustofluidics

## Abstract

While boundary-driven acoustic streaming resulting from the interaction of sound, fluids and walls in symmetric acoustic resonances have been intensively studied in the literature, the acoustic streaming fields driven by asymmetric acoustic resonances remain largely unexplored. Here, we present a theoretical and numerical analysis of outer acoustic streaming flows generated over a fluid–solid interface above which a symmetric or asymmetric acoustic standing wave is established. The asymmetric standing wave is defined by a shift of acoustic pressure in its magnitude, i.e., $S_{0}$, and the resulting outer acoustic streaming is analyzed using the limiting velocity method. We show that, in symmetric acoustic resonances ($S_{0}=0$), on a slip-velocity boundary, the limiting velocities always drive fluids from the acoustic pressure node towards adjacent antinodes. In confined geometry where a slip-velocity condition is applied to two parallel walls, the characteristics of the obtained outer acoustic streaming replicates that of Rayleigh streaming. In an asymmetric standing wave where $S_{0}\neq 0$, however, it is found that the resulting limiting velocity node (i.e., the dividing point of limiting velocities) on the slip-velocity boundary locates at a different position to acoustic pressure node and, more importantly, is shown to be independent of $S_{0}$, enabling spatial separation of acoustic radiation force and acoustic streaming flows. The results show the richness of boundary-driven acoustic streaming pattern variations that arise in standing wave fields and have potentials in many microfluidics applications such as acoustic streaming flow control and particle manipulation.

## 1. Introduction

Acoustic streaming is the steady flow driven by acoustic energy dissipation in a viscous fluid. The acoustic energy dissipation in a fluid could occur from two different mechanisms, dissipation in the boundary layers and attenuation in the bulk of the fluid [1]. The resulting streaming from the former case is known as boundary-driven acoustic streaming, which is usually observed in standing wave fields near walls or suspended objects, while the streaming produced by the latter case is called ‘quartz wind’ or Eckart streaming [2], which is typically observed in the bulk of channels much larger than the acoustic wavelength [3]. Understanding the driving mechanisms of acoustic streaming and its variations is important for the design of acoustofluidic devices to enhance or to suppress its effect for lab-on-a-chip applications [4], such as heat and mass transfer enhancement, microfluidic actuation, sensing, sonoporation and drug delivery, and particle manipulation.

In most micro-acoustofluidic systems of interest where standing waves are typically generated, the acoustic streaming fields are generally dominated by boundary-driven acoustic streaming. As early as 1787, the German physicist Chladni [5] observed that randomly distributed fine particles on a vibrating metal plate could move to the antinodes, which was later studied by Faraday [6], who found that it was due to air currents in the vicinity of the plate, i.e., the boundary-driven acoustic streaming. Theoretical analysis on boundary-driven streaming was initiated by Rayleigh [7], who presented an analytical solution for acoustic streaming between two infinite parallel plates in a one-dimensional (1D) standing wave field. Rayleigh found that in the standing wave direction two streaming vortices per half-wavelength could be generated in each half channel, which is now known as outer acoustic streaming or Rayleigh streaming. After that, a series of modifications have been developed for particular cases [8,9,10,11,12,13,14], which have paved the fundamental understanding of acoustic streaming flows. While classical Rayleigh streaming patterns have been widely studied in the literature, in the last decade, the mechanisms behind some new (i.e., those that cannot be explained by Rayleigh’s classical theory) outer acoustic streaming patterns have been explored, such as the transducer-plane streaming [15,16,17], which generates streaming vortices in planes parallel to the driving boundary, and the modal Rayleigh-like streaming [18], in which vortices have a roll size greater than the quarter wavelength of the main acoustic resonance. These aforementioned analyses (and others) on boundary-driven acoustic streaming, starting from Rayleigh to today, have largely focused on those generated in fluid channels or surfaces of rectangular [19] or circular [20] cross-sections where symmetric acoustic resonances (i.e., those with equal magnitude of acoustic pressure crests and troughs) are excited.

In this work, we investigate the outer acoustic streaming fields near a fluid–solid interface (FSI) by combining the basic theory of acoustofluidics and the limiting velocity method. A generalized standing wave field either symmetric or asymmetric to the acoustic pressure node is considered. We verify numerically the outer acoustic streaming in the symmetric case with the classical theory of Rayleigh streaming, and discuss the outer acoustic streaming patterns in various asymmetric acoustic resonances. Our analysis of outer acoustic streaming in asymmetric acoustic resonances elucidates fundamental physical aspects and further has potential applications in micro- and nanoparticle manipulation.

## 2. Mathematical Model

In micro-acoustofluidics systems of particular interest where standing waves are typically excited, the acoustic streaming field that has received more attention than any other is that which arises from acoustic dissipation near FSIs, which, in most cases, could disturb the movements of particles (especially those of nano size) induced by the acoustic radiation force. Here, we aim to analyze and compare this type of acoustic streaming driven by symmetric and asymmetric acoustic resonances. The basic acoustic streaming equations have been presented in the Appendix A.

We assume that the time variation of each of the first-order acoustic quantities is sinusoidal with ω, and let $p_{1}$ (and the components of $u_{1}$) be represented as the real part of a complex value with time factor $e^{i\omega t}.$ Based on this relation, we consider here a generic 1D standing wave field in the x-direction, where the distribution of acoustic pressure is given by

(1) $$p_{1}=A_{0}\sin\left(kx\right)+S_{0},$$

here, k=2π/λ is the wave number with λ being the acoustic wavelength, $S_{0}$ (with $\left|S_{0}\right|\;\leq A_{0}$) is the magnitude of pressure shift, which could be potentially influenced by the geometry of the fluid channel, and $A_{0}$ is the acoustic pressure amplitude for the symmetric case: A symmetric 1D standing wave field that has same magnitude of pressure peaks and troughs is obtained for $S_{0}=0$.

Here, outside the boundary layer, the first-order acoustic velocity is irrotational and has only an x-component. Following the acoustic pressure distribution described in Equation (1), the x-component first-order acoustic velocity field can typically be determined using the linearized Euler’s equation, i.e., $\rho_{0}\partial u_{1}/\partial t+\nabla p_{1}=0$, and is given by

(2) $$u_{1}=\frac{iA_{0}}{\rho_{0}c_{0}}\cos\left(kx\right),$$

where $c_{0}$ is the sound speed in fluid. This equation is applicable to conditions where the magnitude of the acoustic velocity is small compared with the sound speed in the fluid, i.e., $\left|u_{1}\right|\ll c_{0}$.

Here, for a 1D standing wave in the x-direction of fluid channel, since $v_{1}$, $w_{1}$ and their derivatives are zero, the expression for the x-component limiting velocity (Equation (A12)) reduces to

(3) uL=−14ωReu1du1*dx+u1*2+idu1dx,

and the y-component limiting velocity $v_{L}\equiv 0$. For a pure imaginary expression of acoustic velocity derived in Equation (2), Equation (3) further reduces to $u_{L}=-\left(3/4\omega \right)u_{1}^{*}\partial u_{1}/\partial x$, from which it can be seen that the direction of $u_{L}$ is that in which the magnitude of $u_{1}$ decreases. Moreover, it also indicates that it is the product of the acoustic velocity and its spatial gradient rather than either of them that determines the magnitude of limiting velocity.

Hence, by substituting Equation (2) into (3), the limiting velocity that drives the outer acoustic streaming field for the generalized 1D standing wave case presented in Equation (1) is obtained, which follows

(4) $$u_{L}=-\frac{3A_{0}^{2}}{8\rho_{0}^{2}c_{0}^{3}}\sin\left(2kx\right).$$

It is interesting to notice that the limiting velocity that drives the outer acoustic streaming field is independent of the pressure shift $S_{0}$.

## 3. Results and Discussion

**Figure 1:**
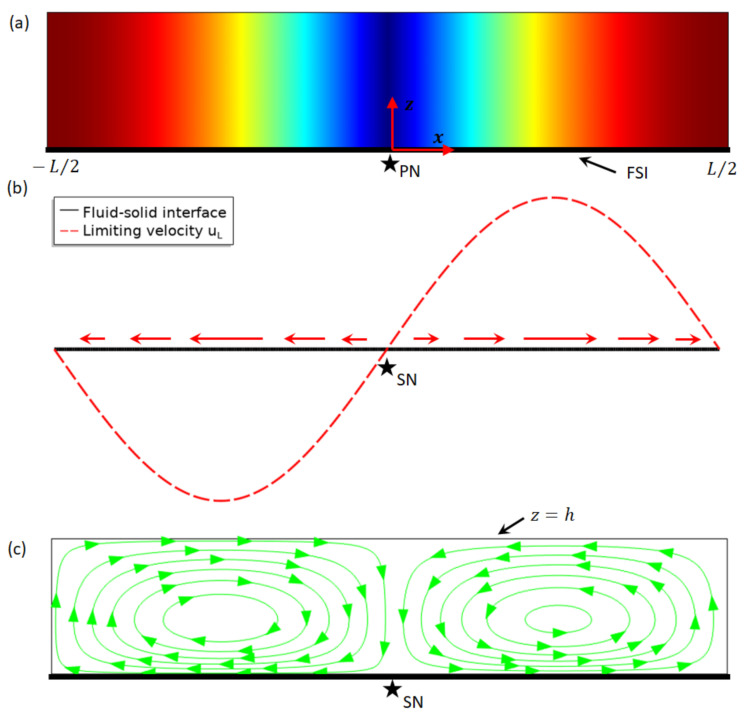
Acoustofluidic fields in a symmetric one-dimensional acoustic resonance over a fluid–solid interface (FSI). (**a**) Acoustic pressure magnitude $\left|p_{1}\right|$ for $S_{0}=0$ over the FSI in a one-dimensional standing wave field (red and blue for maximum and 0, respectively); (**b**) the limiting velocity field $u_{L}$ (see Equation (4)) over the FSI; and (**c**) outer acoustic streaming in a confined fluid driven by the limiting velocities of a half-wavelength standing wave. PN and SN represent locations of acoustic pressure node and acoustic streaming node, respectively.

We now take up the outer acoustic streaming flows associated with a 1D standing wave over a FSI, assuming a slip-velocity condition at the boundary. As shown in Figure 1a, a FSI located at z=0 was considered. We let the first-order acoustic pressure field be given by Equation (1) in the near field of the FSI in the range −L/2<x<L/2 (with λ=2L and x=±L/2 are periodic conditions), which is constant in the z-direction.

The special case $S_{0}=0$ was firstly considered. Without a pressure magnitude shift, as plotted in Figure 1a, it presents a symmetric standing wave with equal magnitude of pressure crests and troughs. The corresponding limiting velocity distribution over the FSI was plotted in Figure 1b. It can be seen that the limiting velocity (and the outer acoustic streaming presented below) and the acoustic pressure share the same location of nodes (denoted by PN and SN, respectively). On the slip-velocity boundary ($u_{bnd}\cdot t=u_{L}$, where the subscript in $u_{bnd}$ indicates the velocity on the boundary), the limiting velocity points from the pressure node to the adjacent antinodes (see arrows). As a result, continuous outer streaming flows could be driven by the limiting velocities near the slip-velocity boundary and for mass conservation (i.e., the total amount of fluid is constant) return flow must occur and vortices could be formed in a confined chamber. Figure 1c shows the outer acoustic streaming vortical flow when a slip condition ($u_{bnd}\cdot n=0$) was applied to the top boundary z=h. Two vortices per half acoustic wavelength were generated in the standing wave direction (i.e., x), while the streaming vortex pattern in the z-direction is dependent on the boundary condition in the far field in the z-direction (e.g., z=h), which could be a nonslip ($u_{bnd}=0$), slip, slip-velocity or symmetric condition, determined by the configuration of the real experimental acoustofluidic device. For example, in a rectangular channel which contains another slip-velocity boundary at z=2h (or a symmetric condition at z=h), two outer acoustic streaming vortices (symmetric to z=h) could be generated between the two parallel slip-velocity boundaries; that is, by symmetry a similar outer streaming pattern to that presented in Figure 1c exists in the other half of the channel (in the range h<z<2h). This type of boundary-driven acoustic streaming generated between two parallel walls which are at z=0 and z=2h in a symmetric 1D standing wave field is the well-known Rayleigh streaming.

**Figure 2:**
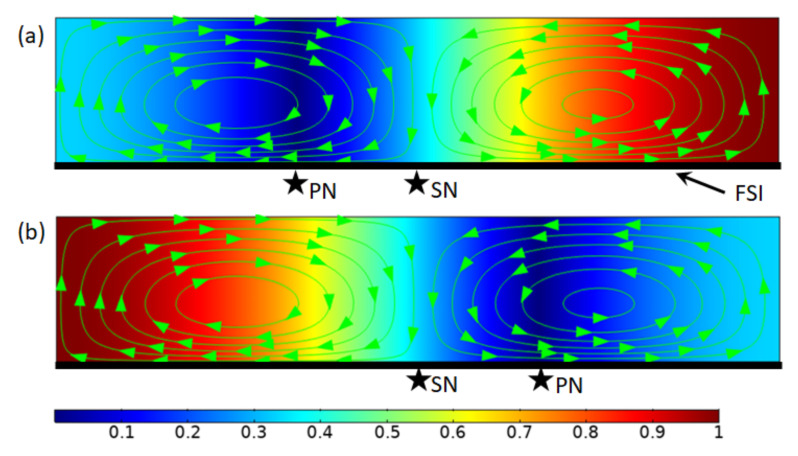
Outer acoustic streaming due to one-dimensional asymmetric acoustic resonances over a fluid-solid interface (FSI). (**a**) $S_{0}=A_{0}/2$; and (**b**) $S_{0}=-A_{0}/2$. The background colors plot normalized acoustic pressure magnitudes and the streamlines show outer acoustic streaming in a confined fluid driven by the limiting velocities over the FSI. PN and SN denote locations of acoustic pressure node and acoustic streaming node, respectively.

However, for the asymmetric cases, i.e., $S_{0}\neq 0$, it was found that the acoustofluidic effects and their relations could be vastly different. As shown in Figure 2, two cases with, respectively, $S_{0}=A_{0}/2$ and $S_{0}=-A_{0}/2$ are presented and compared. It can be seen that the resulting outer acoustic streaming (solved with slip-velocity boundary at z=0 and a slip condition at the top boundary z=h) is identical for these two cases. Moreover, although still two outer acoustic streaming vortices are generated in the half-wavelength in the x-direction, it is interesting to notice that the acoustic streaming node (i.e., the dividing point for limiting velocities) for both cases stays at the channel center rather than at the pressure nodes, different from the situation seen in symmetric acoustic resonances. This, however, is easy to understand from the expression of the limiting velocity; that is, the explanation lies in the fact, to be demonstrated in more detail later, that the magnitude of pressure shift $S_{0}$ is unrelated to the distribution of the x-component limiting velocity (see Equation (4)), which thus has no effect on the outer acoustic streaming pattern.

**Figure 3:**
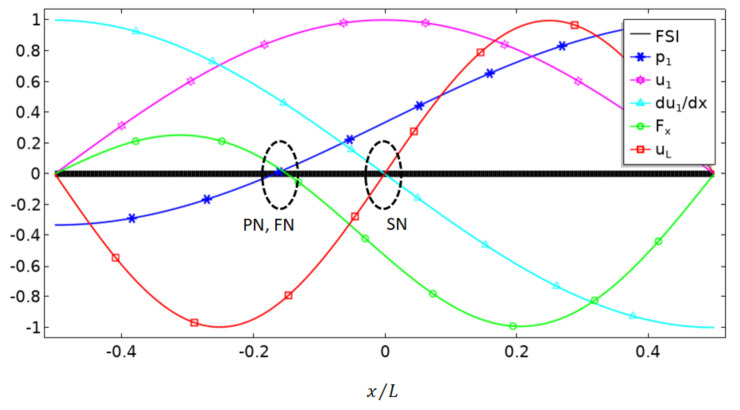
Distributions of the normalized acoustofluidic fields in a one-dimensional asymmetric half-wavelength standing wave field (for $S_{0}=A_{0}/2$, see Equation (1)) over a fluid–solid interface (FSI), including the acoustic pressure $p_{1}$ (asterisk-line), x-component acoustic velocity $u_{1}$ (star-line) and its derivative to x (triangle-line), x-component acoustic radiation force $F_{x}$ (circle-line), and the limiting velocity $u_{L}$ (square-line). PN, FN and SN indicate positions of nodes of pressure, acoustic radiation force and limiting velocity (or acoustic streaming) on the slip-velocity boundary, respectively.

Figure 3 plots the distributions of various acoustofluidic fields for a particular case $S_{0}=A_{0}/2$, from which we aim to obtain further insight into the mechanism of boundary-driven acoustic streaming and to illustrate why the location of limiting velocity nodes (or outer streaming nodes) is independent of $S_{0}$ in asymmetric acoustic resonances and how it could affect acoustophoresis of particles in a microfluidic channel. The square-line in Figure 3 plots the distribution of normalized limiting velocity in −L/2<x<L/2, showing that its node (value of zero or dividing point) locates at x=0 (i.e., the center of the slip-velocity boundary), which is explained by the term $\sin\left(2kx\right)$ in its expression. Moreover, it is worthy of note that, as shown in Equation (4), this distribution is independent of magnitude of pressure shift and thus is valid for any value of $S_{0}$. This relation can also be obtained from alternative explanations. Since the derivative of $p_{1}$ with respect to x does not contain the term $S_{0}$, both $u_{1}$ (star-line in Figure 3) and $du_{1}/dx$ (triangle-line in Figure 3) are independent of $S_{0}$. The limiting velocity $u_{L}$, which is proportional to the product of $u_{1}$ and $du_{1}/dx$ (see Equation (3)), is zero when any of these two values reaches zero, and thus we have $u_{L}\equiv 0$ at the center x=0 for zero value of $\sin\left(kx\right)$ in $du_{1}/dx$ (marked as SN in Figure 3).

For the acoustic pressure and velocity distributions presented in Figure 3, the corresponding distribution of x-component acoustic radiation force on suspending particles in water over the FSI was plotted in Figure 3 (circle-line), which was calculated using the Gorkov potential [21],

(5) $$F_{x}=\frac{\partial }{\partial x}\left\{\frac{4\pi r^{3}}{3}\left[\frac{3\left(\rho_{p}-\rho_{0}\right)}{2\rho_{p}+\rho_{0}}\bar{E_{kin}}-\left(1-\frac{\rho_{0}c_{0}^{2}}{\rho_{p}c_{p}^{2}}\right)\bar{E_{pot}}\right]\right\},$$

where r (≪λ) is particle radius, $\bar{E_{kin}}=\rho_{0}{\left|u_{1}\right|}^{2}/4$ and $\bar{E_{pot}}={\left|p_{1}\right|}^{2}/\left(4\rho_{0}c_{0}^{2}\right)$ are the time-averaged kinematic and potential energy density, $\rho_{p}$ and $\rho_{0}$ are the density of the particle and fluid, $c_{p}$ and $c_{0}$ are the sound speed in particle and fluid. As shown, the acoustic radiation force tends to move rigid particles towards the acoustic pressure node, which is similar to the case seen in symmetric acoustic resonances. As known, in an acousto-microfluidic channel, the combination of acoustic radiation force and acoustic streaming-induced drag force plays a vital role in particle acoustophoresis. As discussed above, we see spatial separation of acoustic radiation force and limiting velocities (or outer acoustic streaming flows) in asymmetric acoustic standing wave fields, indicating that it should be possible to build up different asymmetric acoustic resonances to obtain different groups of acoustic radiation force and acoustic streaming-induced drag force.

## 4. Conclusions

We have demonstrated here that outer acoustic streaming flows driven by 1D symmetric and asymmetric standing wave fields near a FSI could behave very differently. The results for symmetric acoustic resonances replicate the characteristics of Rayleigh streaming: Nodes of acoustic pressure and limiting velocity (or outer acoustic streaming) share same locations; that is, on a slip-velocity boundary which drives circulations of outer acoustic streaming the direction of limiting velocity always goes from acoustic pressure nodes to adjacent antinodes. For asymmetric acoustic resonances, it is interesting to notice that acoustic streaming nodes on a slip-velocity boundary always locate at the channel center wherever the acoustic pressure nodes are, enabling spatial separation of acoustic pressure nodes and acoustic streaming flows. Asymmetric acoustic resonances could potentially be generated in fluid channels where neighbor FSIs are not orthogonal (e.g., channels of triangular or quadrilateral cross-section). In a fluid channel of irregular cross-section, the acoustic pressure field may slightly vary in other directions (e.g., z) of the channel, which, however, will not greatly affect the resulting acoustic streaming field. This is based on the fact that it is the gradient of the acoustic velocity in the main standing wave direction that determines the distribution of limiting velocities (and thus the location of acoustic streaming vortices and the streaming velocity magnitudes), as described in Equations (A12) and (A13). Therefore, although it is assumed here a 1D standing wave in the x-direction of the fluid channel (which is perhaps the simplest case), the results presented here are applicable for the analysis of outer acoustic streaming fields generated in asymmetric resonances in other more complex structures or geometries. We anticipate that with precisely engineered positions of nodes of acoustic pressure and streaming in microfluidic systems, desired combinations of acoustic radiation and acoustic streaming-induced drag forces in a fluid could be designed and obtained, which could provide versatile means for acoustofluidic manipulation of micro- and nano-particles.

## Appendix A

We assume a homogeneous isotropic fluid in which the pressure, density and velocity at any point in the fluid are given by p, ρ and u, and the continuity and momentum equations for the fluid motion are, respectively,

(A1) $$\frac{\partial \rho }{\partial t}+\nabla \cdot \left(\rho u\right)\;=0,$$

(A2) $$\rho \left(\frac{\partial u}{\partial t}+u\cdot \nabla u\right)=-\nabla p+\mu \nabla^{2}u+\left(\mu_{b}+\frac{1}{3}\mu \right)\nabla \nabla \cdot u,$$

where μ and $\mu_{b}$ are the dynamic and bulk viscosity coefficients for the fluid, respectively.

To obtain, respectively, the acoustic and streaming equations, let us now introduce the perturbation theory. It approximates that a steady second-order acoustic streaming flow (absent without acoustic actuation) is superposed on the first-order acoustic velocity field. Using the perturbation method, we can write the fluid density, pressure, and velocity in the form

(A3) $$\rho =\rho_{0}+\rho_{1}+\rho_{2}+\cdots ,$$

(A4) $$p=p_{0}+p_{1}+p_{2}+\cdots ,$$

(A5) $$u=u_{1}+u_{2}+\cdots ,$$

where the subscripts 0, 1 and 2 represent the static (i.e., absence of acoustic excitation), first-order and second-order quantities, respectively.

Substituting Equations (A3)–(A5) into Equations (A1) and (A2) and taking the first-order into account, the continuity and momentum equations become

(A6) $$\frac{\partial \rho_{1}}{\partial t}+\rho_{0}\nabla \cdot u_{1}=0,$$

(A7) $$\rho_{0}\frac{\partial u_{1}}{\partial t}=-\nabla p_{1}+\mu \nabla^{2}u_{1}+\left(\mu_{b}+\frac{1}{3}\mu \right)\nabla \nabla \cdot u_{1}.$$

To obtain equations for acoustic streaming, we keep terms up to second order and take the time average of the continuity and momentum equations. Equations (A1) and (A2) are then turned into

(A8) $$\nabla \cdot \bar{\rho_{1}u_{1}}+\rho_{0}\nabla \cdot \bar{u_{2}}=0,$$

(A9) $$-\rho_{0}\bar{u_{1}\nabla \cdot u_{1}+u_{1}\cdot \nabla u_{1}}=-\nabla \bar{p_{2}}+\mu \nabla^{2}\bar{u_{2}}+\left(\mu_{b}+\frac{1}{3}\mu \right)\nabla \nabla \cdot \bar{u_{2}},$$

where the upper bar $\bar{X}$ indicates time-average of X. The left-hand-side of Equation (A9) represents the Reynolds stress force [1], which is generated by absorption of acoustic energy due to the viscous effects of fluids and is determined once the first-order acoustic velocity $u_{1}$ is known.

In fluid channels of most acousto-microfluidic devices where standing waves are generated, the acoustic streaming field is generally dominated by boundary-driven acoustic streaming (when the Eckart type streaming [2] contribution is ignored), which results from the acoustic energy dissipation in the thin boundary layer due to the presence of a nonslip condition at the FSI. Furthermore, in most acoustofluidic devices working at MHz frequencies, the thickness of boundary layer (i.e., size of inner acoustic streaming vortices), expressed by $\delta_{v}=\sqrt{2\nu /\omega }$ with $\nu =\mu /\rho_{0}$ and ω being the kinematic viscosity of fluid and angular frequency, is typically much smaller than the channel dimensions (for example, $\delta_{v}\approx 0.53$ µm for 1 MHz frequency), and thus usually only the acoustic streaming fields outside the boundary layer (i.e., the outer acoustic streaming) are of interest.

Outside the boundary layer, $\bar{\rho_{1}u_{1}}$ is zero because $\rho_{1}$ and $u_{1}$ differ in phase π/2 for plane standing waves; Equations (A8) and (A9) can thus be simplified to

(A10) $$\nabla \cdot \bar{u_{2}}=0,$$

(A11) $$-\nabla \bar{p_{2}}+\mu \nabla^{2}\bar{u_{2}}=0.$$

The Reynolds stress force term has also been dropped from Equation (A11) because, in most cases, outside the boundary layer it can only set up hydrostatic stresses, but cannot cause flow rotation in the bulk of the fluid in the absence of attenuation. These equations can be applied to effectively solve the outer acoustic streaming fields when the limiting velocity (the streaming velocity just outside the boundary layer, which is a function of the first-order acoustic velocity field) is applied as a slip-velocity boundary condition.

The analysis of limiting velocities outside the boundary layer was initiated by Schlichting [8] and later modified by Nyborg [10] and Lee and Wang [12] to more generalized cases. Typically, in three-dimensional Cartesian coordinates (xyz), the limiting velocities are simply expressed by [15]

(A12) uL=−14ωReu1du1*dx+v1du1*dy+u1*2+i∇·u1−2+3idw1dz,

(A13) vL=−14ωReu1dv1*dx+v1dv1*dy+v1*2+i∇·u1−2+3idw1dz,

where $u_{L}$ and $v_{L}$ are the x- and y-components of limiting velocities over a flat surface that is normal to z, ω is the angular frequency, the asterisk ∗ represents the complex conjugate, $i=\sqrt{-1}$ is the imaginary unit, and $u_{1}$, $v_{1}$ and $w_{1}$ denote, respectively, the x-, y- and z-components of the acoustic velocity vector $u_{1}$.

Ignoring the inner acoustic streaming, the modelling of outer acoustic streaming by limiting velocities is known as the limiting velocity method, which is more computationally efficient than the Reynolds stress method [19] and is suitable for three-dimensional numerical modelling of boundary-driven acoustic streaming fields. In the last decade, using the limiting velocity method, in symmetric acoustic resonances various types of boundary-driven acoustic streaming patterns such as the classical Rayleigh streaming [22], modal Rayleigh-like streaming [18] and transducer-plane streaming [17] in thin-layer glass capillaries and Rayleigh-like streaming patterns in phononic crystals [23,24,25] have been modelled and elucidated through numerical simulations.
